# 

**Published:** 1817-09

**Authors:** 


### CORRESPONDENCE.

The Editors return thanks to Dr. Quarrier for his very interesting Case of Fractured Cranium, which will be inserted in the ensuing Number of the Journal. Mr. Rolfe's Communication will also appear in our next.

Dr. Davis's valuable Table and Report are unavoidably deferred till our ensuing Number.

A very interesting Case of Hydrophobia has just occurred at Portsmouth; which, with some remarkable appearances on dissection, will probably appear next month in this Journal.

The Editors have received the very remarkable and instructive Case of Thoracic Inflammation, by Mr. J. Simpson, Assistant Surgeon of the 36th Regiment, which shall shortly appear.

The promised Communication of Mr. M'Leod, lately returned from the Embassy to China, will be thankfully received by the Editors.

The "*Authentic Cases*" are thankfully received; and our much-valued Correspondent is informed, that the Porte-Feuille and Miscellanies are ever open to such Communications; consequently, a separate head for that purpose would be unnecessary.

Dr. J. will answer the last interesting letter of Dr. D****** by a private conveyance, in the course of ten days. Dr. D—— will see some of his hints embodied in the current Number of the Journal.

We shall receive with unfeigned pleasure the promised materials for a biographical sketch of the lamented Mr. Allan Burns.

Review of Mr. Good's Nosology will appear in our next.

Several highly important foreign works, French, German, and Italian, are under Review.

Dr. Wilson Philip's Experimental Inquiry into the Laws of the Vital Functions, &c; and Dr. Dewar's Account of an Epidemic Small-pox, at Cupar, have been received.

